# Stress and Psychopathology Reduction in Pregnant Women through Online Cognitive Behavioural Therapy during COVID-19: A Feasibility Study

**DOI:** 10.3390/bs11070100

**Published:** 2021-07-09

**Authors:** Jose A. Puertas-Gonzalez, Carolina Mariño-Narvaez, Borja Romero-Gonzalez, Maria Isabel Peralta-Ramirez

**Affiliations:** 1 Mind, Brain and Behaviour Research Center (CIMCYC), 18071 Granada, Spain; puertasjose@ugr.es (J.A.P.-G.); caromarinar1@gmail.com (C.M.-N.); mperalta@ugr.es (M.I.P.-R.); 2 Personality, Assessment and Psychological Treatment Department, Faculty of Psychology, University of Granada, 18071 Granada, Spain; 3 Psychology Department, Faculty of Education, Campus Duques de Soria, University of Valladolid, 42004 Soria, Spain

**Keywords:** cognitive behavioural therapy, pregnancy, resilience, stress, online therapy

## Abstract

Background: The global pandemic has affected the psychological health of the population, including pregnant women. Due to the difficulty of offering conventional therapies to reduce stress in this population, studies are needed to show the effect of online therapies. Therefore, the objective was to test the effect of online cognitive behavioural therapy in pregnant women during the pandemic on the main variables of stress and psychopathology. Methods: The sample consisted of 16 pregnant women who participated in a weekly cognitive behavioural intervention for 8 weeks. Prenatal concerns, general stress, stress vulnerability, resilience and psychopathology were assessed. Results: The results show a reduction in prenatal concerns, perceived stress, stress vulnerability and psychopathology, as well as an increase in resilience. Conclusions: Online cognitive behavioural intervention may be effective in pregnant women, so it is important to conduct a randomised controlled trial to certify these findings.

## 1. Introduction

The appearance of an outbreak of a new pneumonia disease caused by coronavirus (COVID-19) in December 2019 has led to drastic changes in the population’s way of life, carrying new challenges to be faced, as well as changes in social relations and personal liberties [1].

In this way, a population that has been particularly affected are pregnant women, who have presented an increase in their stress levels, depression and/or anxiety during pregnancy [2,3]. This increase can have serious consequences on maternal and infant health, as years of studies have demonstrated the close relationship between stress and the development of depression in pregnancy and postpartum depression, increased risk of preeclampsia and hypertension, increased risk of miscarriage, need for instrumental deliveries, low foetal weight and premature birth [4,5,6]. In addition to these negative consequences, it was recently shown that women who have gave birth during the pandemic also experienced a low satisfaction with their delivery, higher levels of stress during childbirth and increased symptoms of postpartum depression [7]. For that reason, different psychological interventions have emerged with the aim of reducing the levels of stress in pregnant women, such as mindfulness-based programs (MBPs) [8,9], relaxation techniques such as progressive muscle relaxation and guided imagery [10] and even sport-based interventions such as yoga [11,12]. However, evidence-based medicine highlights cognitive behavioural therapy (CBT) as a treatment to reduce a wide range of psychopathology and stress [13]. CBT aims to modify distorted cognitive thoughts and interpretations of reality that lead to disturbing emotions and feelings [14,15]. CBT for stress coping has shown great results in reducing stress in pregnant women [16], or in reducing anxiety or depression levels [17]. Thus, promoting such interventions during pregnancy can help reduce the negative effects of stress and improve maternal and infant health. Thus, promoting such interventions during pregnancy can help reduce the negative effects of stress and improve maternal and infant health.

The pandemic has been a major barrier to psychological treatments, as they must now be carried out online in many cases. However, some studies highlight the applicability of this type of treatment, since video calls also allow for face-to-face interaction [18]. In addition, the patient’s self-efficacy may increase, as success is less likely to be attributed solely to the work of the therapist [19].

Therefore, it is necessary to test the feasibility of CBT for stress management in times of pandemic, conducted telematically, in order to verify whether a subsequent randomised controlled trial is appropriate. Thus, the aim of this study was to test the effect of online cognitive behavioural stress management therapy (o-CBT) in pregnant women, applied during the COVID-19 pandemic, on the main variables of psychological stress and psychopathology.

## 2. Materials and Methods

### 2.1. Participants

This study included a total of 16 pregnant women. The inclusion criteria were: pregnant women with a good command of spoken and written Spanish and with an internet connection to follow the sessions. Exclusion criteria included having suffered from a psychological illness in the past year or at present, or suffering from a current medical illness. Given that some research recommends 12 participants as a minimum for pilot studies [20], and taking into account our study objectives, design and intervention, research suggests that a sample size of 16 participants is reasonable [21,22].

All women who agreed to participate read an information sheet and gave their consent through an online questionnaire platform. The protocol of this study was reviewed and approved by the Biomedical Ethics Research Committee of the Junta de Andalucía (internal code 0401-M1-17). Moreover, this study followed the guidelines of the Helsinki Declaration (AMM, 2008) and the Good Clinical Practice Directive (Directive 2005/28/EC) of the European Union.

### 2.2. Instruments

First of all, participants were asked to answer some questions regarding sociodemographic and obstetric information. After that, the following instruments were used for the psychological evaluation of the participants:

- The Symptom Checklist-90-Revised (SCL-90-R) [23,24]. This is a 90-item scale with 5 points, ranging from 0 (never) to 4 (extremely). This instrument is used to evaluate nine different measurements: somatization, obsession–compulsion, interpersonal sensitivity, depression, anxiety, hostility, phobic anxiety, paranoid ideation and psychoticism. There are also seven additional items on the scale distributed among 3 global indexes of distress: the GSI, which measures overall psychological distress; the PSDI, which is used to calculate the intensity of symptoms; and Positive Symptom Total, used to measure the number of self-reported symptoms. The scores are converted to percentiles (0–100) according to the author’s instructions. Percentiles ≥ 75 represent clinical symptoms in any of the subscales of this instrument. The nine dimensions show a reasonable reliability, with a Cronbach’s alpha for internal consistency of 0.81.
- Pregnancy Distress Questionnaire (PDQ) [25,26]. This is a 12-item scale that measures pregnancy-specific stress related to maternal concerns about pregnancy, such as medical problems, labour and delivery, physical signs and symptoms, bodily changes and the baby’s health. A 5-point Likert-type scale is used to collect responses where 4 = very much and 0 = not at all. The Cronbach’s alpha reliability coefficient is 0.71.
- Perceived Stress Scale (PSS) [27,28]. The PSS collects data on people’s perceptions of general stress over the previous month. It is made up of 14 elements that are rated on a 5-point Likert scale. (0 = never, 1 = almost never, 2 = once in a while, 3 = often, 4 = very often). Scores range from 0 to 56 (higher scores represent higher levels of stress). The Spanish Cronbach’s reliability alpha coefficient is 0.81.
- Stress Vulnerability Inventory (IVE) [29]. It consists of 22 items that assess a person’s proclivity to be affected by perceived stress. It has a Yes/No answer format. Items receiving an affirmative answer add 1 point. The range of scores on the scale is 0 to 22, with higher scores corresponding to greater vulnerability to stress. The scale is highly reliable, with a Cronbach’s alpha of 0.87.
- Connor Davidson Resilience Scale (CD-RISC) [30,31]. It reflects the ability to cope with stressful situations such as changes, personal problems, illness, pressure, failure and feelings of pain. The CD-RISC-10 consists of 10 items, and a Likert scale with 5 response options ranging from 0 (“almost never”) to 4 (“almost always”). It has a Cronbach’s alpha reliability coefficient of 0.86.

### 2.3. Procedure

The participants were recruited through the dissemination in social networks of news published in different newspapers and media for two weeks. These news items included the contact information of a researcher from the team, so that interested pregnant women could get in touch. All women who wished to participate read the study information sheet and gave their consent through an online questionnaire platform. The objective was to carry out two groups of 8–10 people each. Finally, 25 participants were interested in participating, of which 6 did not meet the inclusion criteria. The remaining 19 took part in two pilot groups to test the efficacy and feasibility of the online version of the treatment, of which 16 completed the intervention.

Participants attended 8 consecutive weekly o-CBT sessions of 1.5 to 2 h duration with two trained psychologists with a master’s degree of 900 h of training in psychological treatment. o-CBT was performed through Google Meet. In addition, the psychologists had conducted the same intervention on several occasions in face-to-face format prior to the pandemic. A Google Forms link was administered, through which participants completed the pre-treatment assessment, composed of the previously described assessment instruments.

The intervention was adapted from a previous treatment program [32], whose efficacy has been demonstrated in pregnant women [16]. The program consists of 8 sessions with the following content: (1) psychoeducation: what stress is, its characteristics, how to identify stressors, how to react to them and what are its effects; (2) deactivation strategies (thematic imagination and diaphragmatic breathing); (3) cognitive restructuring: cognitive distortions; (4) cognitive restructuring: irrational beliefs; (5) alternative ways to control your thoughts: self-instructional training and time management; (6) training in social skills: assertiveness, fundamental assertive rights, saying no and requesting a change of behaviour; (7) relationship between anger and stress: emotional self-control; and (8) optimism and sense of humour summary.

Each intervention followed the same structure and the same guidelines to assume the standardisation of the contents. At the beginning of the session, participants received an email with the link to connect to the virtual session, the documents needed to work during the session, the behavioural self-report and the tasks to work on at home that week related to the topic of the session. In order to maximize the benefits of the online intervention, it was recommended at the beginning of each session to activate the camera and to use headphones and microphone. In addition, all participants were encouraged to participate in the sessions with different actions, such as: talking about their experiences, expressing difficulties about the tasks between sessions, role-playing, etc.

At the conclusion of treatment, the evaluation instruments described above were administered again.

### 2.4. Data Analysis

First, a descriptive and frequency analysis was carried out to check the distribution of the sample in the main sociodemographic and obstetric history variables.

Secondly, the Shapiro–Wilk test was used to test the normality of the data. In the case of the SCL-90-R scores, since they did not present a normal distribution, the Wilcoxon W test was performed for two related samples (pre and post). The rest of the scores (EEP, PDQ, IVE, CDRISC) met the assumption of normality and therefore a Student’s *t* test was performed for two related samples (pre and post).

Finally, to calculate the effect size of the statistically significant changes of the parametric variables that were analysed with the Student’s *t* test, Cohen’s d was used (small effect size > 0.20, medium effect size > 0.50 and large effect size > 0.80). The calculation is based on Borenstein’s formulas [33] for calculating the effect size in a *t*-analysis for dependent samples. In the case of non-parametric variables, the formula proposed by Cohen [34] (*r* = Z/√N) was used, where [Z] is the absolute value of the Z score and [N] is the total number of observations. This effect size comprises values from 0 to 1, for which Cohen (1988) recommended the following interpretations: *r* > 0.10 small effect size, *r* > 0.30 medium effect size and *r* > 0.50 large effect size.

Analyses were performed using the Statistical Package for the Social Sciences 26.0 for Windows (SPSS, Armonk, NY, USA).

## 3. Results

### 3.1. Sample Description

A total of 16 pregnant women took part in the pilot study, with a mean age of 36.63 years (SD = 3.36) and who were between 6 and 31 weeks of pregnancy (M = 20.50 weeks gestation; SD = 7.81). All of them were Spanish and were married or cohabiting with their partner (*n* = 16; 100%). In addition, 87.5% (*n* = 14) had a university degree. The rest of the variables can be consulted in Table 1.

### 3.2. Changes in Psychological Variables at the End of Therapy in Pregnant Women

First, Student’s *t*-analyses for dependent samples showed statistically significant differences in: pregnancy-specific stress (*t* = 4.446; *p* ≤ 0.001) with a medium effect size (d = 0.671); perceived stress (*t* = 3.243; *p* ≤ 0.005) with a high effect size (d = 0.909); stress vulnerability (*t* = 3.982; *p* ≤ 0.001) with a low effect size (d = 0.348); and resilience (*t* = −2.643; *p* ≤ 0.01) with a medium effect size (d = 0.608). As can be seen in Table 2, after finalizing the treatment women showed decreased scores in these variables.

Regarding the non-parametric analyses performed for the scores in the different dimensions of the SCL-90-R, statistically significant differences were found in obsessions and compulsions (Z = −2.556; *p* ≤ 0.01) with a high effect size (r = 0.638); anxiety (Z = −3.201; *p* ≤ 0.01) with a high effect size (r = 0.80); phobic anxiety (Z = −2.003; *p* ≤ 0.05) with a medium effect size (r = 0.501); psychoticism (Z = −2.202; *p* ≤ 0.05) with a medium effect size (r = 0. 550); and in the general scales of global severity index (Z = −2.030; *p* ≤ 0.05), positive symptoms (Z = −2.207; *p* ≤ 0.05) and positive distress index (Z = −2.336; *p* ≤ 0.05). In addition, it should be noted that the study population showed clinical scores (greater than 70) on the SCL-90-R subscales of obsessions and compulsions, depression, anxiety and psychoticism prior to treatment. These data and the means for each measure can be found in Table 2. As can be seen, after treatment, not only was there a decrease in the psychopathological symptom scores in these women, but also in depression, anxiety and psychoticism, in which they went from clinical scores to normal scores.

## 4. Discussion

The aim of this study was to verify the effect of o-CBT on stress management in pregnant women. In order to achieve it, a 2 month online intervention (eight sessions) was implemented with a total of 16 women.

Firstly, it was proven that women who completed the intervention had a significant reduction in their stress levels, both in perceived stress and pregnancy-specific stress. The main component of o-CBT is stress management; thus, the online version of the therapy supports the results found by previous authors in a randomised controlled trial [16]. Additionally, this reduction could have direct repercussions on maternal and foetal health, since pregnancy-specific stress has been considered as a powerful predictor of negative outcomes [35].

Additionally, there was a reduction in the vulnerability to stress and an increase in resilience scores. These results are highly relevant, taking into account that not only the problematic factors, such as stress levels, reduce, but it goes one step forward, increasing resilience, which could also carry improvements in coping mechanisms, self-esteem and general wellbeing [36,37].

In addition, there was found to be a decrease in various psychopathological symptoms in the women who participated in the therapy. On one hand, obsession and compulsion levels and phobic anxiety decreased; these are characteristic symptoms related to the actual pandemic situation. The need to maintain extreme hygiene to avoid contagion, or the fear of vertical transmission of the virus to the foetus, could underlie the presence of this symptomatology [38,39]. Reducing these levels is a matter of extreme importance, since obsessions and phobias, maintained during pregnancy, can shunt into intense fears that disable the pregnant woman, occasionally resulting in a phobia towards childbirth, which in addition generates low satisfaction towards it, a higher chance of suffering a traumatic childbirth and even the need to require an instrumentalised procedure [6,40,41].

Furthermore, anxiety is highly related to the stress suffered and the phobic anxiety previously described. Around 8% of pregnant women report suffering symptoms of anxiety [42], which are related to the development of severe psychopathological disorders after childbirth, such as postpartum depression, a higher chance of a preterm birth and internalizing problems on the offspring [4,43].

Finally, psychoticism symptoms also reduced after therapy. Other authors have stated that this symptomatology increases in pregnant women, probably due to an evolutionary aspect of pregnancy, where the mother considers external agents as a threat [44]. During pandemics such as the one we are enduring, this symptomatology can increase, because in order to avoid contagion there is an aversion to meet new people or maintain a social life [45]. o-CBT offers the possibility of increasing social support without having to be in direct contact with their peers, which could be related to the diminishment of this symptom. Additionally, on average, the participants started the therapy in the second trimester and finished in the first weeks of the third trimester, where the psychological state of pregnant women typically worsens [4]. As it has been verified, o-CBT palliated this psychological worsening in the sample, helping to improve the psychological health during the third trimester of pregnancy.

Despite the promising findings reported, this study presents a limitation; since this is an applicability study, and does not have a control group, the changes found cannot be related exclusively to the therapy’s efficacy. Moreover, despite following previous research recommendations for sample size [20,21,22], the sample size is quite small, which could affect statistical power. Apart from that, it would be interesting to include working-class women in the future study, to see how stress is reduced in this sample. Therefore, it is of great relevance to continue research of the efficacy of o-CBT, implementing at least one control group. It would also be interesting to include an additional control group centred on social support, in order to guarantee that the changes found are due to the abilities acquired through the therapy and not just the supportive environment where pregnant woman share experiences with their peers.

Preliminary results show an unquestionable improvement in the psychological health of women who participated in the therapy, which is in accordance with the results of a face-to-face version [16]. It is important to raise the fact that as the pregnant women had no psychological illnesses in our sample, this may be a step towards implementing this therapy as a health-promoting measure. Nowadays, these interventions are especially necessary during pregnancy as a means to promote health, in order to avoid the loneliness and abandonment that is produced by a situation such as the pandemic we are living in and to provide the pregnant women with strategies that help them palliate the negative psychological effects caused by the pandemic.

## Figures and Tables

**Table 1:** Sociodemographic variables and obstetric information of the sample.

		Therapy Group (*n* = 16) M(SD)/%
Sociodemographic variables		
Age		36.63 (3.36)
Weeks of gestation	T_0_	20.50 (7.81)
	T_1_	28.50 (7.81)
Married/cohabiting	Yes	16 (100%)
Nationality	Spanish	16 (100%)
Education level	High school	2 (12.5%)
	University	14 (87.5%)
Employment situation	Employed	2 (12.5%)
	Part-time employment	1 (6.3%)
	Full-time employment	13 (81.3%)
Obstetric information		
Primiparous	Yes	7 (43.8%)
	No	9 (56.3%)
Pregnancy method	Spontaneous	14 (87.5%)
	Fertility treatment	2 (12.5%)
Wanted pregnancy	Yes	14 (87.5%)
	No	2 (12.5%)
Previous miscarriages	0	9 (56.3%)
	1	6 (37.5%)
	≥2	1 (6.3%)

Note: T_0_ = pre-intervention; T_1_ = post-intervention.

**Table 2:** Differences in psychological measures before and after therapy.

	Measures	T_0_	T_1_	*t*/Z	*p*	d/r
	PDQ	19.88(7.15)	15.25(6.49)	4.446	0.001 **	0.671
	PSS	28.75(5.62)	22.69(7.43)	3.243	0.005 **	0.909
	IVE	9.69(5.49)	7.50(5.98)	3.982	0.001 **	0.348
	CD-RISC	21.19(5.35)	24.63(5.89)	−2.643	0.018 *	0.608
	SOM	61.19(29.02)	53.69(32.48)	−1.616	0.106	0.403
	OBS	86.88(13.20)	78.88(16.50)	−2.556	0.011 *	0.638
	SEN	67.75(30.13)	59.81(30.12)	−1.207	0.227	0.301
	DEP	76.31(22.52)	68.56(27.56)	−1.310	0.190	0.327
	ANS	78.75(18.36)	68.81(22.90)	−3.201	0.001 **	0.801
SCL-90-R	HOS	62.63(28.32)	58.69(32.74)	−0.772	0.440	0.192
	FOB	67.56(31.09)	52.88(36.54)	−2.003	0.045 *	0.501
	PAR	55.00(41.50)	48.94(41.99)	−0.840	0.401	0.209
	PSI	79.94(20.47)	67.75(26.98)	−2.202	0.028 *	0.550
	IGS	79.94(19.23)	68.88(28.13)	−2.030	0.042 *	0.507
	SP	81.56(19.00)	73.19(24.59)	−2.207	0.027 *	0.551
	PSDI	63.69(20.33)	50.00(27.14)	−2.336	0.019 *	0.584

Note: T_0_ = pre-intervention; T_1_ = post-intervention; * = *p* value ≤ 0.05; ** = *p* value ≤ 0.01; PDQ = Pregnancy Distress Questionnaire; PSS = Perceived Stress Scale; IVE = Stress Vulnerability Inventory; CD-RISC = Connor Davidson Resilience Scale; SCL-90-R = The Symptom Checklist-90-Revised; SOM = somatizations; OBS = obsession and compulsion; SEN = interpersonal sensitivity; DEP = depression; ANS = anxiety; HOS = hostility; FOB = phobic anxiety; PAR = paranoid ideation; PSI = psychoticism; IGS = global severity index; SP = positive symptoms; PSDI = positive symptoms distress index.

## Data Availability

Data are available on request to the corresponding author.
